# The Detection of Trace Metal Contaminants in Organic Products Using Ion Current Rectifying Quartz Nanopipettes

**DOI:** 10.1021/acs.analchem.4c00634

**Published:** 2024-04-03

**Authors:** Emer B. Farrell, Fionn McNeill, Alexander Weiss, Dominik Duleba, Patrick J. Guiry, Robert P. Johnson

**Affiliations:** School of Chemistry, University College Dublin, Belfield, Dublin 4, Ireland

## Abstract

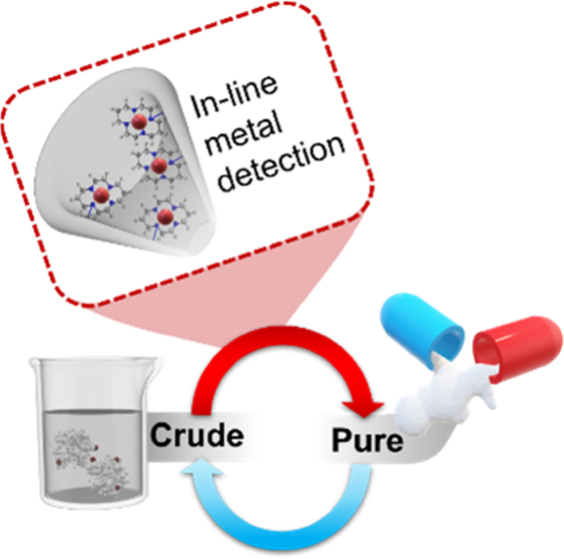

While ion current rectification (ICR) in aprotic solvent has been fundamentally studied, its application in sensing devices lacks exploration. The development of sensors operable in these solvents is highly beneficial to the chemical industry, where polar aprotic solvents, such as acetonitrile, are widely used. Currently, this industry relies on the use of inductively coupled plasma mass spectrometry (ICP-MS) and optical emission spectroscopy (OES) for the detection of metal contamination in organic products. Herein, we present the detection of trace amounts of Pd^2+^ and Co^2+^ using ion current rectification, in cyclam-functionalized quartz nanopipettes, with tetraethylammonium tetrafluoroborate (TEATFB) in MeCN as supporting electrolyte. This methodology is employed to determine the concentration of Pd in organic products, before and after purification by Celite filtration and column chromatography, obtaining comparable results to ICP-MS within minutes and without complex sample preparation. Finite element simulations are used to support our experimental findings, which reveal that the formation of double-junction diodes in the nanopore enables trace detection of these metals, with a significant response from baseline even at picomolar concentrations.

## Introduction

Ion current rectification (ICR) describes the nonohmic current–voltage traces exhibited by asymmetric nanopores, where electrical double layer (EDL) overlap, and resulting tip perm-selectivity, means inequal currents are measured at equal, but opposite, potentials.^1,2^ ICR has been described by a number of models, mostly focused on aqueous systems. Woermann described ICR in relation to ion transference numbers at the tip, with ion enrichment/depletion occurring due to the perm-selectivity of the overlapping EDL, giving rise to high- and low-conductivity states.^3^ Siwy et al.^4,5^ described high- and low-conductivity states arising due to the formation of electrostatic potential ion traps, where EDL overlap occurs. ICR was demonstrated theoretically by Cervera et al.,^6,7^ who solved the Poisson–Nernst–Planck equations to describe ion transport through an asymmetric nanopore. Due to the dependence of ICR on EDL overlap, and hence, nanopore surface charge, it can be employed in sensing applications, where functionalization of the nanopore surface with a probe molecule and subsequent change in surface charge upon binding of an analyte give rise to a change in ICR.^8,9^ ICR-based nanopore sensors have been reported for the detection of a range of aqueous analytes, including, but not limited to, small drug molecules,^10−12^ monosaccharides,^13,14^ enzymes,^15^ neurochemicals,^16^ proteins,^17−20^ phosphates,^21^ carbonates,^22^ DNA,^23,24^ pesticides,^25^ antioxidants,^26^ and mycotoxins.^27,28^

This methodology has also been reported for the detection of aqueous metals, most commonly through the functionalization of the nanopore wall with chelating metal ligands. Examples include tannic acid for Cu^2+^ and Fe^3+^ detection,^29^ macrocyclic dioxotetraamines for Hg^2+^ detection,^30^ and imidazole for Co^2+^ detection.^31^ Cr^3+^ has also been detected through chelation to the surface hydroxyl and carboxyl groups of a PET membrane, requiring no surface functionalization.^32^ Similarly, nonimmobilized polyglutamic acid probes have been employed for the detection of Cu^2+^.^33^ Other surface functionalization procedures, with larger molecules, have also been reported, including polyelectrolytes for the detection of Cu^2+^,^34^ peptide aptamers for the detection of Ni^2+^,^35^ and single-stranded DNA for the detection of Hg^2+^.^36^

The development of nanopore metal sensors operable in organic solvent is beneficial to the fine chemical industry, where metal catalysts are heavily utilized.^37^ Synthetic chemistry predominantly occurs in organic solvents including alcohols, dichloromethane (DCM), and acetonitrile (MeCN),^38^ and developing sensors operable in these solvents allows for in-line and/or rapid detection of trace metals. Currently, ICP-MS and OES are used end-of-line to detect trace metals in organic compounds, which requires digestion of solid and liquid samples in concentrated nitric acid prior to analysis and the use of He gas, an expensive and at-risk elemental resource (or Ar gas for ICP-OES). Nanopore sensors offer a simple, low-cost alternative with miniaturization capabilities, allowing for in-line or batch contaminant detection.

A number of fundamental studies have explored the particularities of ICR in aprotic solvent and the influence of local solvent ordering. Since the surface silanol groups of quartz remain protonated in these solvents, the origin of nanopore surface charge, hence, ICR, is more complex. Plett et al.^39^ and Yin et al.^40^ proposed that the surface charge of nanopores filled with aprotic solvent arises due to the dipole orientation of the solvent molecules along the neutral nanopore wall. Further work by Polster et al.^41^ and Souna et al.^42^ described the formation of a lipid-like bilayer of MeCN at a solid silica interface, and the resulting effective surface potential arising as electrolyte ions interact with it. Remarkably, Silva et al.^43^ reported that this organization of solvent is dependent on chirality, with enantiopure propylene carbonate (PC) exhibiting a lower positive surface potential than racemic PC. In our previous work, we reported the unusual behavior of bare quartz nanopipettes in aprotic solvent as a function of decreasing supporting electrolyte concentration, showing that under specific electrolyte conditions, accumulation of aprotic solvent and the subsequent formation of double-junction diodes within the nanopore gives rise to unexpectedly high rectification ratios.^44^ We believe that these unique behaviors can be exploited for sensing applications, in particular, the double-junction diode amplification effect, and may allow us to achieve lower detection limits than possible in water.

**Figure 1:**
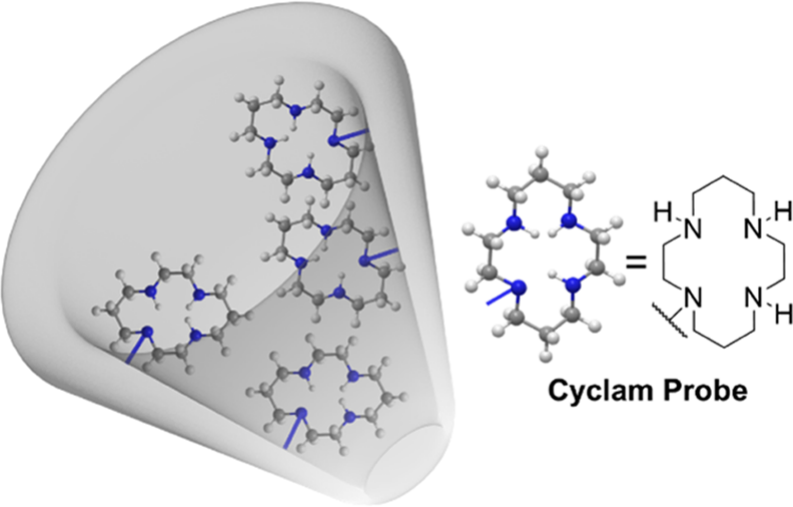
Schematic of the cyclam-functionalized quartz nanopipette tip employed in this work for the detection of Co^2+^ and Pd^2+^.

To the best of our knowledge, ICR in aprotic solvent has only been reported in fundamental studies and has never been exploited for sensing applications. Herein, we present a cyclam-functionalized quartz nanopipette (Figure 1) capable of exploiting the double-junction diode effect to identify trace metal ions at the picomolar concentration range in the aprotic solvent acetonitrile. In addition, we show that these nanopipettes can be used to analyze organic reactions that employ homogeneous Pd catalysts, with aliquots for analysis taken before, and after, purification by Celite filtration and column chromatography, with comparable results to ICP-MS.

## Experimental Methods

### Materials and Reagents

Quartz capillaries (0.7 mm I.D., 1 mm O.D., Sutter Instruments) were used in the fabrication of the quartz nanopipettes. The electrolyte employed in organic ICR experiments was tetraethylammonium tetrafluoroborate (99%, Alfa Aesar) dissolved in acetonitrile (99.9%, Fisher Scientific). Nanopipette radii were measured using potassium chloride (99%, Acros Organics) dissolved in Milli-Q water with Ag/AgCl wires (prepared using Ag wires (99.9%, Merck)) as working and reference electrodes. Pt wires (99.9%, Merck) were used as electrodes in organic electrolyte systems. 1,4,8,11-Tetraazacyclotetradecane (98%, Sigma-Aldrich), 3-iodopropyltrimethoxysilane (95%, Sigma-Aldrich), potassium carbonate (99.5%, Fluorochem), acetonitrile (99.9%, Fisher Scientific), and pentane (99%, Fisher Scientific) were used to synthesize silylated cyclam. The Pd-catalyzed reactions were carried out with rigorous exclusion of air and moisture under an inert atmosphere of nitrogen in flame-dried glassware with magnetic stirring, unless otherwise stated. N_2_-flushed plastic syringes were used to transfer air- and moisture-sensitive reagents. Oxygen-free nitrogen was obtained from BOC gases. 4-Bromotoluene, phenylboronic acid pinacol ester, Pd(PPh_3_)_4_, and (*R*,*R*)-ANDEN-phenyl Trost ligand were purchased from Sigma-Aldrich and used as received. Anhydrous 1,4-dioxane was obtained from commercial sources and used as received. Tris(dibenzylideneacetone)palladium(0) chloroform adduct was prepared via the method of Ananikov.^45^ In vacuo refers to the evaporation of solvent under reduced pressure on a rotary evaporator. Flash column chromatography was performed using 40–63 μm, 230–400 mesh silica gel.

All current–voltage traces were measured using a Biologic SP-200 potentiostat fitted with an ultralow current option. Measurements were performed with a filter bandwidth of 50 kHz, and a moving average filter (window size of 11 points) was applied after measurement using EC-Lab software to filter the noise numerically.

### Fabrication of Nanopipettes

Nanopipette fabrication was carried out using a Sutter P-2000 micropipette puller with 5 tunable parameters heat (H), filament (F), velocity (V), delay (D), and pull (P). The following program was employed to fabricate 50 nm nanopipettes (Line 1: H700, F4, V20, D170, and P0, Line 2: H680, F4, V50, D170, and P200) from 0.7 mm quartz capillaries.

### Characterization of Nanopipettes

Nanopipette radii were determined by recording current–voltage traces using 0.1 M KCl electrolyte in deionized water. Nanopipettes were backfilled with electrolyte, and a Ag/AgCl wire working electrode was inserted. The nanopipettes were placed in a bulk electrolyte bath containing a Ag/AgCl wire reference electrode such that the tip was submerged, and current–voltage traces were measured. The applied potential was swept from −1 to 1 V with respect to the reference electrode, at a scan rate of 0.1 V s^–1^. A linear fit was applied to the resulting CV using EC-Lab software, and the slope was used to calculate the nanopipette radius based on eq 1.^46,47^
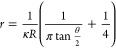
1where κ is the electrolyte conductivity, θ is the cone angle, and *R* is the nanopipette resistance. By inserting resistance, the inverse of conductivity (obtained from the slope of the CV), the nanopipette radius (*r*) can be determined, assuming a constant cone angle between nanopipettes and excluding the effect of nanopipette wall surface charge. The calculated radii are shown in Figure S4a. The calculated radii were verified by using electron microscopy. SEM images were recorded with a Zeiss ultra plus at an accelerating voltage of 2 kV using the SE2 detector, and STEM images were recorded using a Zeiss Sigma300 FEG SEM at 10 kV acceleration using a STEM InLens detector. Representative images are shown in Figure S4b,c.

### ICR Measurements

**Figure 2:**
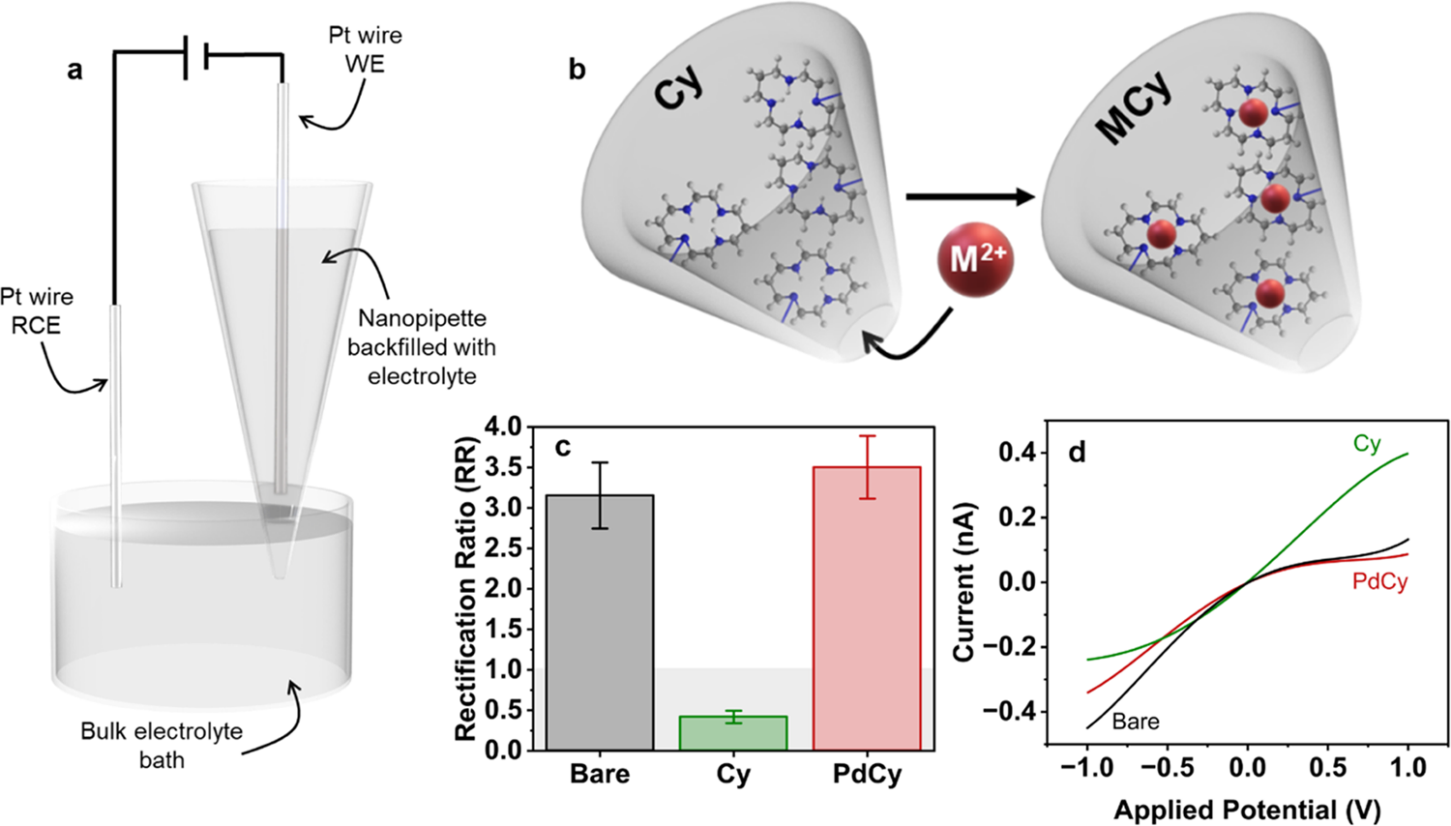
(a) Two-electrode experimental setup employed for ICR measurements. (b) Schematic showing M^2+^ ions binding in cyclam-functionalized nanopipette tips. (c) Rectification ratio and (d) CV measurements (polynomial fit) of bare (black) and cyclam-functionalized (green, Cy) quartz nanopipettes and the response of cyclam-functionalized nanopipettes to 0.1 nM PdCl_2_ dissolved in the bulk electrolyte (red, PdCy). All CVs are measured in 0.2 mM TEATFB in MeCN, at a scan rate of 0.1 V s^–1^, from −1 to +1 V, using a Biologic SP-200 potentiostat. All nanopipettes have a radius of ∼50 nm.

Nanopipettes were backfilled with 0.2 mM tetraethylammonium tetrafluoroborate (TEATFB) in acetonitrile (MeCN). A Pt wire working electrode was inserted into the nanopipettes, which were placed in a bulk electrolyte bath of the same concentration containing a Pt wire reference electrode (Figure 2a). For metal sensing, CVs were first measured in a neat 0.2 mM TEATFB/MeCN bulk electrolyte bath, followed by the measurement in a 0.2 mM TEATFB/MeCN bulk electrolyte bath spiked with metal samples. Current–voltage traces were measured using a Biologic SP-200 potentiostat with an ultralow current probe. The applied potential was swept from −1 to 1 V with respect to the reference electrode, at a scan rate of 0.1 V s^–1^.

### Synthesis of 3-(1,4,8,11-Tetraazacyclotetradecane) Propyltrimethoxysilane

1,4,8,11-Tetraazacyclotetradecane (0.3 g) and potassium carbonate (0.051 g) were combined in MeCN (15 mL), and the resulting dispersion was heated to reflux. Once refluxing, 3-iodopropyltrimethoxysilane (0.15 mL) in MeCN (6 mL) was added dropwise. The dispersion was refluxed for 16 h under N_2_. Upon completion, the reaction was cooled to room temperature, and MeCN was removed under vacuum. To the crude solid was added pentane to remove insoluble impurities. Finally, pentane was removed under vacuum, yielding a yellowish solid (0.124 g).

### Functionalization of Quartz Nanopipettes with 3-(1,4,8,11-Tetraazacyclotetradecane)propyltrimethoxysilane (Silyl Cyclam)

Silyl cyclam was dissolved in MeCN to a concentration of 0.8 mg mL^–1^. Quartz nanopipette tips were submerged in 300 μL of the silyl cyclam solution for 30 min, after which they were dipped in MeCN for cleaning and backfilled with 0.2 mM TEATFB/MeCN. The nanopipettes were placed on a hot plate at 75 °C for 30 min for thermal filling, after which CV measurements were carried out.

### Suzuki–Miyaura Cross-Coupling Employing [Pd(PPh_3_)_4_] for the Electrochemical Analysis of Pd Content

To a flame-dried 50 mL Schlenk, 4-bromotoluene (85.5 mg, 0.5 mmol, 1 equiv), phenylboronic acid pinacol ester (112.2 mg, 0.55 mmol, 1.1 equiv), and Pd(PPh_3_)_4_ (5.8 mg, 0.005 mmol, 1 mol %) were added. The reactants were dissolved in propan-1-ol (8 mL) to which a sodium carbonate solution (2 mL, 1 M, 4 equiv) was added. The reaction was stirred for 2 h, after which a 1 mL aliquot was taken and the solvent removed in vacuo (Sample R1-1). The solvent was removed from the remaining material in vacuo, giving a dirty brown oil, which was redissolved in DCM and filtered through Celite, washing with 100 mL of DCM. Finally, the remaining solution was concentrated to 10 mL, after which a 1 mL aliquot was taken and the solvent was removed in vacuo (Sample R1-2). This remaining material was concentrated and tested by ^1^H NMR spectroscopy. The resulting spectrum was clean and matched the literature data; therefore, no further purification was carried out.

### Decarboxylative Asymmetric Allylic Alkylation of a Sterically Hindered α-Allyl-α-aryl Lactone for the Electrochemical Analysis of Pd Content

In a 10 mL, flame-dried Schlenk tube, Pd_2_(dba)_3_·CHCl_3_ (5.9 mg, 5.0 mol %), (*R,R*)-ANDEN-phenyl Trost ligand (12 mg, 13.0 mol %), and α-aryl-β-oxo allyl ester (40 mg, 1 equiv) were dissolved in 1,4-dioxane (0.04 M) (2.85 mL). The reaction mixture was stirred under a N_2_ atmosphere at 40 °C for 18 h. After this time, a 0.5 mL aliquot was taken from the reaction mixture, and the solvent was removed in vacuo (Sample R2-1). The reaction mixture was filtered through a Celite plug and washed with DCM (3 mL). The remaining solvent was removed in vacuo, and the crude residue was purified by flash column chromatography (10% EtOAc in cyclohexane increasing to 25%). The product was collected as an off-white oil (Sample R2-2). The product was analyzed by ^1^H NMR spectroscopy and matched literature data,^48^ so no further purification was carried out.

### Finite Element Simulations

COMSOL Multiphysics 6.0 was used to solve Poisson–Nernst–Planck and Navier–Stokes equations, using the following physics modules; transport of diluted species (tds), electrostatics (es), and creeping flow (spf). The basic nanopipette geometry was modeled as 2D axisymmetric with a pipet height of 5 μm, a pipet radius of 50 nm, and a half-cone angle of 10°, as shown in Figure S1. The bulk electrolyte was modeled as a square of width 2.5 μm. A region for finer meshing of the electrical double layer (EDL) with a width of 5 nm was input, as well as a small rectangular region at the nanopipette tip with a height of 5 nm. The nanopipette wall was assumed to have a width of 2 nm. Mesh refinement was performed until there was no change in RR with a decreasing element size. The meshing is shown in Figure S1. Boundary conditions were applied at the nanopipette wall, interior bulk solution, and exterior bulk solution, as shown in Table S1 and Figure S1. The dielectric constant for MeCN was taken to be 37.5. The diffusion coefficients were based on reported values, where NEt_4_^+^ was 0.96 × 10^–9^ m^2^ s^–1^ and BF_4_^–^ was 0.82 × 10^–9^ m^2^ s^–1^.^49^

The Nernst–Planck equation was used to simulate the flux of ions arising from diffusion, migration, and convection:
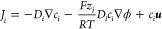
2where *J*_i_ is the flux of an ion, *D*_i_ is the diffusion coefficient of an ion, *c*_i_ is ion concentration, *z*_i_ is ion charge, *R* is the ideal gas constant, *T* is temperature, *ϕ* is the electric potential, and ***u*** is the fluid velocity.

The Poisson equation was used to solve the distribution of the electric field:
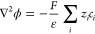
3where ϕ is the electric potential, ε is the dielectric permittivity, *z*_i_ is ion charge, and *c*_i_ is ion concentration.

The Navier–Stokes equation was used to solve fluid velocity and pressure distribution:
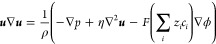
4where ***u*** is the fluid velocity, ρ is the solvent density, η is the solvent viscosity, *p* is the applied pressure, *z*_i_ is the ion charge, *c*_i_ is the ion concentration, and ϕ is the electric potential.

Capillary filling of bare nanopipettes was simulated using the Cahn–Hilliard phase field method. This employs the following physics modules: phase filling (pf) and creeping flow (spf). The boundary conditions and relevant equations are provided in the Supporting Information.

## Results and Discussion

### Cyclam Probes Are Immobilized on the Inner Surface of Quartz Nanopipettes

**Scheme 1:**
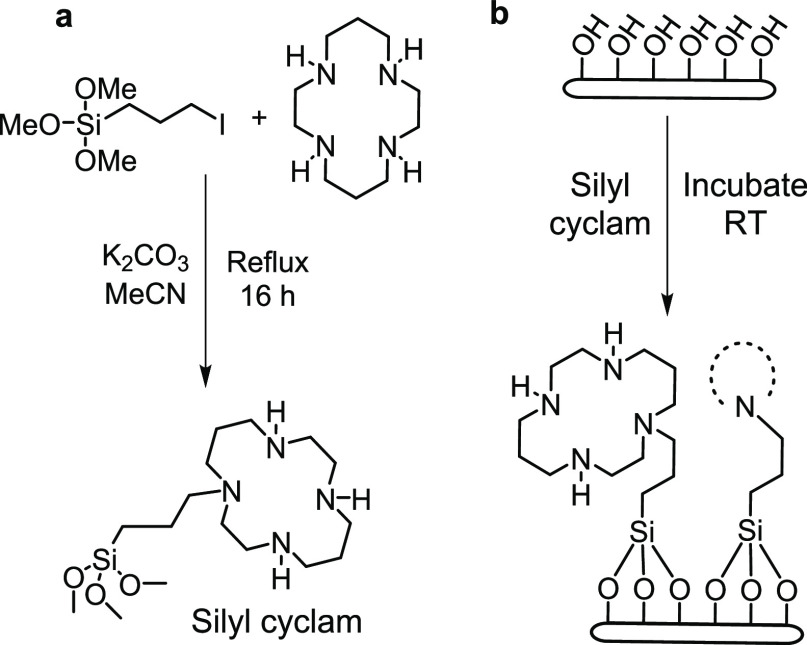
Synthesis of Silyl Cyclam (a) and Immobilization to Quartz Nanopore Wall (b)

The attachment of cyclam probe moieties to the internal surfaces of a quartz nanopore (Scheme 1) was achieved through the in-house synthesis of silyl cyclam and its subsequent immobilization to the bare internal glass surface. Synthesis of the silyl cyclam, based on a previous scheme reported by Dubois et al.,^50,51^ was achieved by overnight reflux of cyclam, potassium carbonate (K_2_CO_3_), and (3-iodopropyl)trimethoxysilane in MeCN.

Silyl cyclam was immobilized on quartz nanopipettes following a simple, one-step dipping procedure. Bare quartz nanopipette tips were submerged in a solution of silyl cyclam (0.8 mg mL^–1^) for 30 min, after which they were dipped in MeCN to remove excess silyl cyclam, and backfilled with 0.2 mM tetraethylammonium tetrafluoroborate (TEATFB) in MeCN.

In 0.2 mM TEATFB in MeCN, bare quartz nanopipettes with a radius of ∼50 nm are negatively rectifying, meaning their rectification ratio is greater at negative than positive potential, as described in our previous work.^44^ The rectification ratio (RR) is used to quantify the extent and direction of the ICR. It is defined in eq 5 as the current at negative potential (*I*^–^) divided by the current at positive potential (*I*^+^). A nanopore with negative ICR has an RR greater than 1, while a nanopore with positive ICR has an RR less than 1.
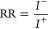
5

Upon immobilization of cyclam to the nanopore wall, ICR switches from negative to positive (Figure 2c), due to a change in surface charge distribution along the nanopore wall, which is explained using finite element simulations, as shown in Figure 5.

### Cyclam-Functionalized Nanopipettes Are Highly Responsive to Metal Ions

Addition of metal dichloride salts to the external bulk electrolyte bath and subsequent binding of these metal ions to the immobilized cyclam probe groups (Figure 2b) give rise to significant changes in ICR (Figure 2c,d). In the presence of M^2+^, the rectification ratio becomes more negative and increases from ∼0.5 to, at maximum change, ∼3.5 (in the presence of 0.1 nM PdCl_2_). Each cyclam-functionalized nanopipette is measured with and without the presence of metal ions, and the percentage change is calculated according to eq 6.
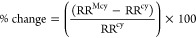
6where RR^Cy^ and RR^MCy^ denote the RR of a cyclam-functionalized nanopipette measured in the absence and presence of metal ions, respectively.

Due to the universal nature of the immobilized cyclam probe molecule, the metal ions Pd^2+^ and Co^2+^, which are widely used in the catalysis of organic reactions, were chosen for study. Calibration curves were generated for each metal by serial dilution of a stock electrolyte/metal solution (of known metal concentration), from 100 to 0.001 nM.

**Figure 3:**
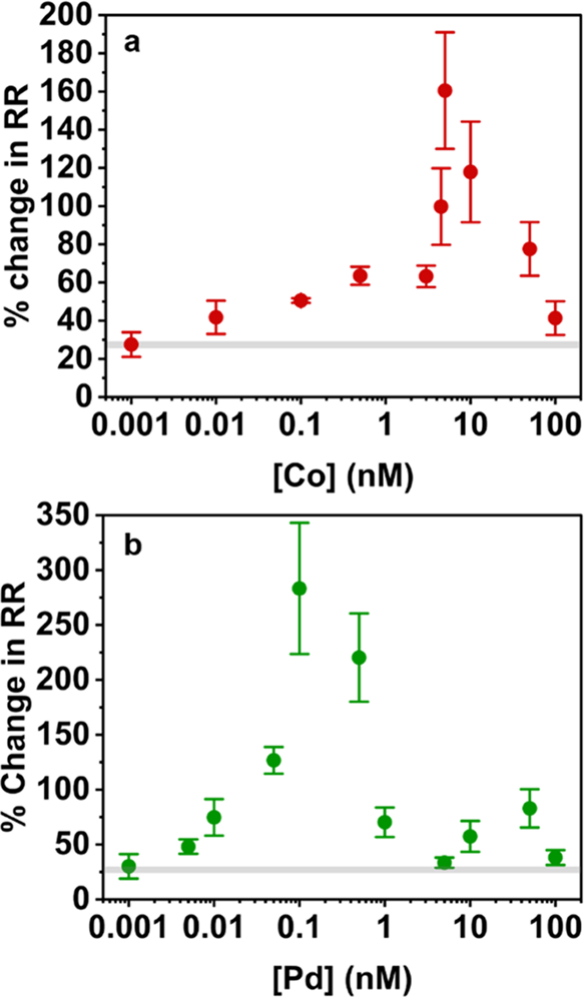
% change in rectification ratio (RR) as a function of decreasing MCl_2_ concentration in the bulk electrolyte bath, for (a) CoCl_2_ and (b) PdCl_2_. All CVs are measured in 0.2 mM TEATFB in MeCN, at a scan rate of 0.1 V s^–1^, from −1 to +1 V, using a Biologic SP-200 potentiostat. All nanopipettes have a radius of ∼50 nm. CVs are measured directly after the addition of MCl_2_ to the bulk electrolyte bath, with no incubation period. The gray shaded area shows the control range, in the presence of no MCl_2_.

Interestingly, the percentage response initially increases or oscillates as a function of decreasing metal concentration until reaching a point where a “classical” decrease in signal with decreasing analyte concentration is observed. Each metal generates a characteristic curve shape, varying in degree of oscillation and position of maximum percentage change (Figure 3).

Co^2+^ exhibits the maximum percentage change at a CoCl_2_ concentration of 5 nM, after which an exponential decrease in response in the range of 3 to 0.001 nM CoCl_2_ is observed (Figure 3a). Pd^2+^ oscillates toward a maximum % change at 0.1 nM PdCl_2_, after which the response decreases exponentially in the range of 0.1–0.001 nM PdCl_2_ (Figure 3b). These results indicate that cyclam-functionalized nanopipettes act as binary response sensors for Co^2+^ and Pd^2+^ over a substantial concentration range with any change in RR outside of the control range confirmation of the presence of a concentration of M^2+^ greater than 0.001 nM. Furthermore, the “classical” regions of decreasing response (3–0.001 and 0.1–0.001 nM, respectively) can be used for quantitative analysis of appropriately diluted samples.

The cyclam probes used in this work do not have specificity for individual metal ions and, as observed at certain concentrations, produce similar responses for Co^2+^ and Pd^2+^. However, based on the different quantitative exponential range of each calibration curve, we believe the metal present can be identified through serial dilution of the sample. Furthermore, cyclam moieties can be adapted to induce selectivity, through the introduction of electron-donating or -withdrawing groups, and additional chelating pendant arms.

It is important to note that the measurements described in this work are carried out in HPLC grade MeCN (not anhydrous) under atmospheric conditions, which was also used in our previously reported fundamental study.^44^ This is because the experimental complexity introduced by the requirement for dry, inert conditions is not feasible for the applications that we envisage for our technology. Plett et al.^39^ and Yin et al.^40^ have previously shown that ICR in dimethylformamide (DMF), 1,2-dichloroethane (DCE), and propylene carbonate (PC) is affected by water content. However, Plett et al.^39^ saw a lesser impact in PC, which was attributed to the high degree of adsorbed solvent ordering. Additionally, Souna et al.^42^ discussed the remarkable stability of MeCN ordering at a solid silica interface, which is persistent even in the presence of substantial amounts of water. For this reason, we do not believe that water absorption affects the experimental results described in this work. Furthermore, analyte detection is immediate with no required incubation period, meaning conditions remain constant, and control experiments in the absence of M^2+^ (represented by the gray shaded area in Figure 3) show only a minor change in RR.

Reusability studies were carried out to determine the stability of the cyclam-functionalized nanopipettes, and the binding of the metal ions was found to be reversible. These results are summarized in Figure S6. In short, after exposure to M^2+^, a temperature-aided regeneration returns the nanopipettes to their initial state, and subsequent exposure to the same M^2+^ concentration results in the same percentage change as initially observed. After 3 cycles a change in RR of the initial cyclam-functionalized nanopipettes to ∼1 occurs; however, the response to M^2+^ still remains the same. We believe this change in RR arises due to a partial loss of surface-bound cyclam with repeated exposure to elevated temperatures. Despite the fact that no loss of sensor functionality occurs, it remains necessary to identify a regeneration procedure that does not affect the initial state of the cyclam-functionalized nanopipettes.

### Finite Element Simulations Reveal the Importance of Double-Junction Diode Formation in the Measurement of Sensitivity

**Figure 4:**
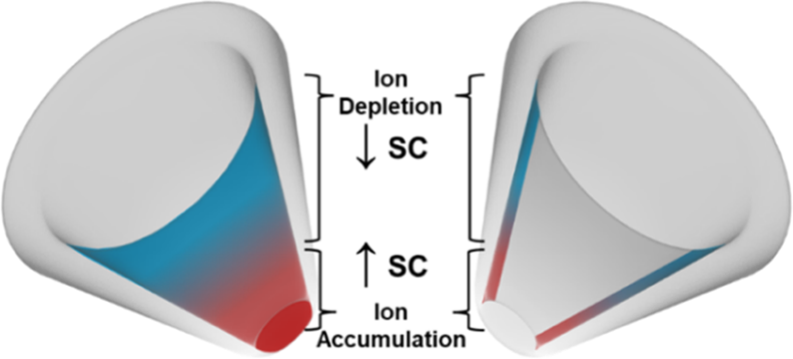
Schematic showing double-junction diode formation in a bare quartz nanopipette in TEATFB/MeCN, when an internal potential is applied.

Finite element simulations were carried out using COMSOL Multiphysics, to solve the Poisson–Nernst–Planck-Navier–Stokes equations to (1) determine the effect of cyclam functionalization on ICR and (2) to investigate its oscillatory response to metal binding. In our previous work,^44^ we described the formation of double-junction diodes in quartz nanopores in TEATFB/MeCN electrolyte solutions. This phenomenon dictates ion transport in aprotic solvent and is important to consider when developing sensors. It is schematically represented in Figure 4, showing bands of higher (and lower) surface charge along the nanopore wall, originating from ion enrichment (and depletion) which gives rise to solvent enrichment (and depletion). This solvent enrichment effect is essentially a feedback loop, with accumulation of solvent causing an increase of ions and an increase of ions leading to an increase in solvent. The position of, and surface charge within, double-junction diodes have a significant effect on ICR. Thus, we anticipated that changes to the double-junction diodes arising due to nanopore functionalization and metal–ligand binding were likely to play a key role in the observed trend in rectification ratio with trace metal concentration.

**Figure 5:**
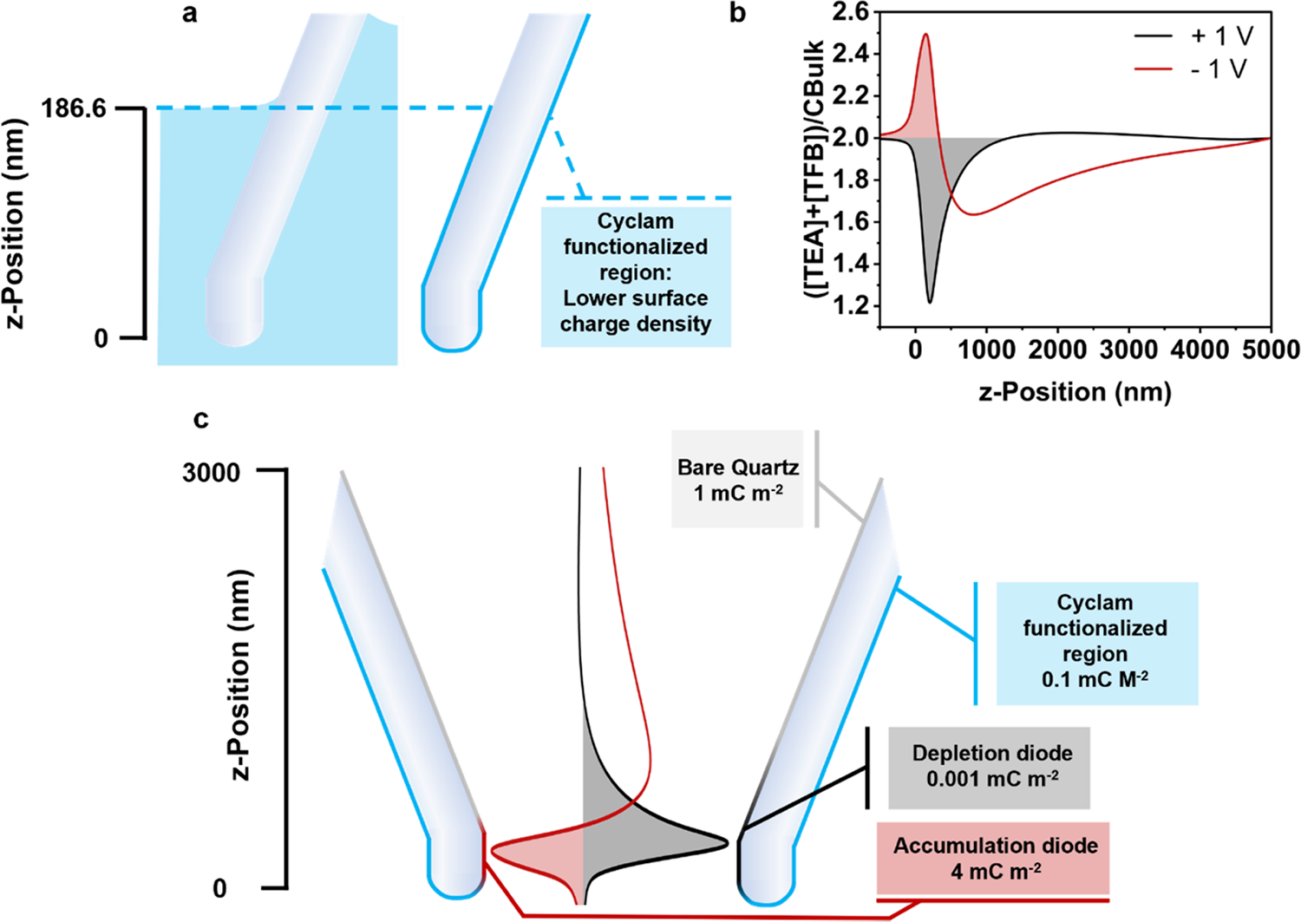
(a) Schematic of the meniscus position of the silyl cyclam solution in the nanopipette tip and the resulting cyclam-functionalized region of the nanopipette. (b) Normalized ion enrichment and depletion curves along the nanopipette *z*-axis, with incorporation of a region of lower surface charge density (0.1 mC m^–2^) up to the meniscus position. (c) Schematic showing incorporation of the accumulation and depletion diodes at +1 and −1 V into the model as regions of higher, and lower, surface charge density. Simulations are calculated in 0.5 mM TEATFB/MeCN due to convergence issues at lower concentrations.

The nature of the dipping technique employed for nanopipette functionalization indicates that the determination of the depth of the solution’s meniscus inside the nanopore is imperative. The meniscus height for a nanopore, with a radius of 50 nm and half-cone angle of 10°, is calculated to be 186.6 nm, using the Cahn–Hilliard equations. A different surface charge density is implemented in the cyclam-functionalized region of the nanopore, while assuming an even surface charge density of 1 mC m^–2^ along the rest of the nanopore wall (Figure 5a). From this, ion enrichment and depletion bands are obtained (Figure 5b) and used to construct double-junction diodes with a series of surface charge density values in the cyclam-functionalized region (Figure 5c). The boundaries of the double-junction diodes are determined as shown in Figures S2 and S3 and are included in the model using step functions. For simplicity, ion accumulation is assumed to give a surface charge density of 4 mC m^–2^, and depletion is assumed to give a surface charge density of 0.001 mC m^–2^. In reality, these surface charge density values would be far more extreme, and vary significantly with differing surface charge density in the cyclam-functionalized region.^44^ However, for the sake of our simulation, which seeks only qualitative agreement, consideration of these factors is not necessary. Our calculations indicate that cyclam functionalization results in a decrease in surface charge, which is likely due to the lower polarity of the N–H bonds in cyclam, as compared to the bare quartz’s O–H surface groups.

**Figure 6:**
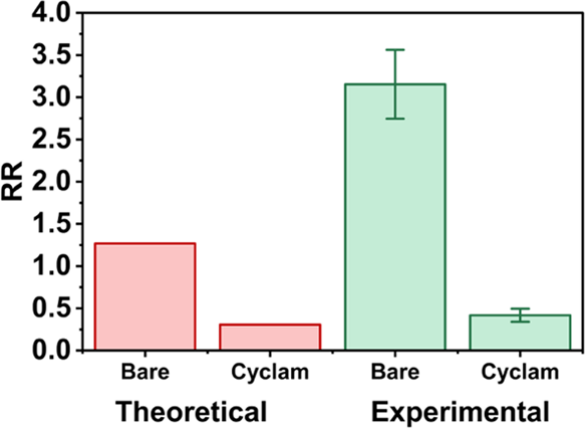
Effect of cyclam functionalization on theoretically and experimentally observed RR, with a surface charge density of 0.1 mC m^–2^ in the cyclam-functionalized region of the nanopipette. All simulations are calculated with inclusion of double-junction diodes (as shown in Figure 5), in 0.5 mM TEATFB/MeCN (due to convergence issues at 0.2 mM TEATFB).

**Figure 7:**
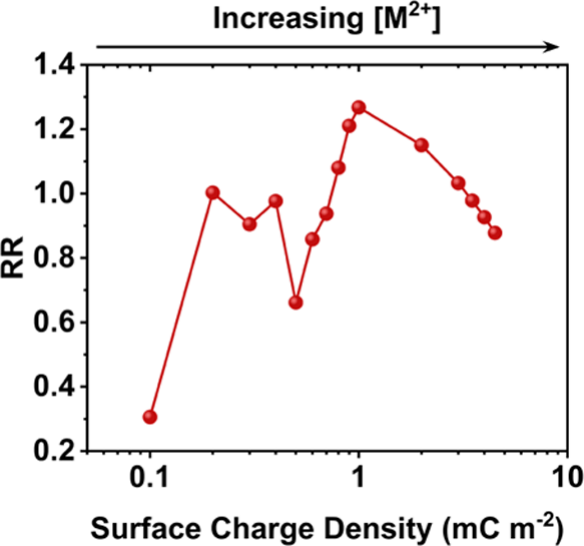
Theoretical results showing the change in RR as a function of increased surface charge density (0.1–4.5 mC m^–2^) in the cyclam-functionalized region of the nanopipette. All simulations are calculated with the inclusion of double-junction diodes (as shown in Figure 5), in 0.5 mM TEATFB/MeCN (due to convergence issues at 0.2 mM TEATFB).

The closest agreement with the experimentally observed RR after cyclam functionalization was reached at a surface charge density of 0.1 mC m^–2^ in the cyclam-functionalized region (Figure 6). Following this, metal binding can be modeled by increasing the surface charge density in the cyclam-functionalized region (due to the presence of M^2+^ ions) and calculating RR using double-junction diodes, as previously described. As shown in Figure 7, an oscillatory response in the RR is observed as the surface charge density in the cyclam-functionalized region is increased, in agreement with our experimental results. This oscillation arises due to the significant changes in ion accumulation and depletion that occur as a function of surface charge density in the cyclam-functionalized region. At low surface charge densities (0.1 mC m^–2^), depletion is observed at positive potential, and accumulation at negative potential (Figure S2a). At surface charge densities from 0.2 to 0.4 mC m^–2^, depletion is present at both positive and negative potentials (Figure S2b). From 0.5 to 1 mC m^–2^, ion accumulation occurs at positive potential, and depletion occurs at negative potential (Figure S2c). Finally, at high surface charge densities (2–4.5 mC m^–2^), depletion again occurs at both positive and negative potential (Figure S2d).

### Pd Concentration in Acetonitrile Solutions of Organic Products before and after Purification Can Be Determined with Minimal Pretreatment

To demonstrate the viability of our sensor in “real world” applications, organic reactions utilizing homogeneous Pd catalysts were performed, and aliquots were taken along various stages of purification to determine the content of Pd remaining in the sample. Our nanopore analysis (which does not require acid digestion or substantial pretreatment) was compared to standard ICP-MS results, to determine its accuracy.

**Scheme 2:**
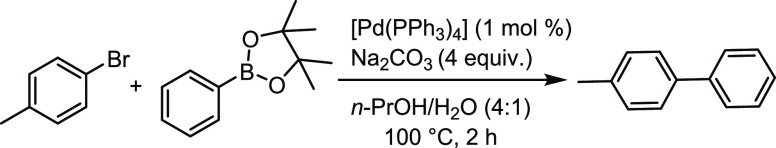
Suzuki–Miyaura Cross-Coupling Reaction Employing Pd(PPh_3_)_4_

**Scheme 3:**
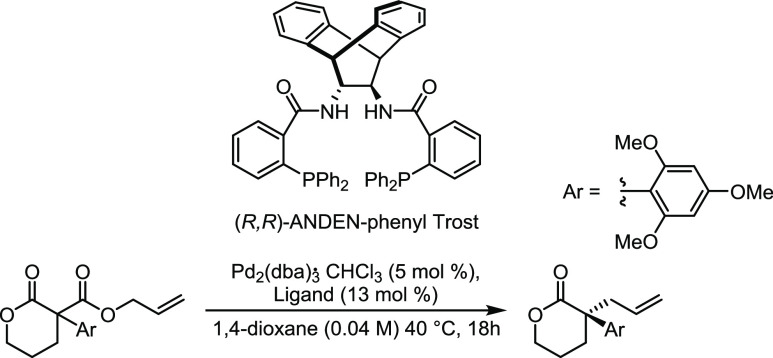
Decarboxylative Asymmetric Allylic Alkylation of an α-Allyl-α-aryl Lactone Employing Pd_2_(dba)_3_ and (*R*,*R*)-ANDEN-Phenyl Trost Ligand

The reactions studied were: (a) a Suzuki–Miyaura cross-coupling reaction employing tetrakis(triphenylphosphine)palladium (Pd(PPh_3_)_4_) (Scheme 2)^52^ and (b) a decarboxylative asymmetric allylic alkylation (DAAA) of an α-allyl-α-aryl lactone employing tris(dibenzylideneacetone)dipalladium (Pd_2_(dba)_3_) in the presence of the chiral (*R*,*R*)-ANDE*N*-phenyl Trost ligand (Scheme 3).^48^ The latter process (DAAA) has been extensively investigated by us as a methodology to prepare sterically hindered, all-carbon quaternary stereocenters possessing α-allyl-α-aryl motifs.^53−56^

The Suzuki–Miyaura reaction is purified by Celite filtration, while the DAAA reaction is purified by column chromatography. Nanopore analysis is carried out as previously described, and the change in RR is calculated for individual nanopipettes measured in 1: neat 0.2 mM TEATFB/MeCN and 2: 0.2 mM TEATFB/MeCN containing dissolved organic product (1 mg 25 mL^–1^).

**Figure 8:**
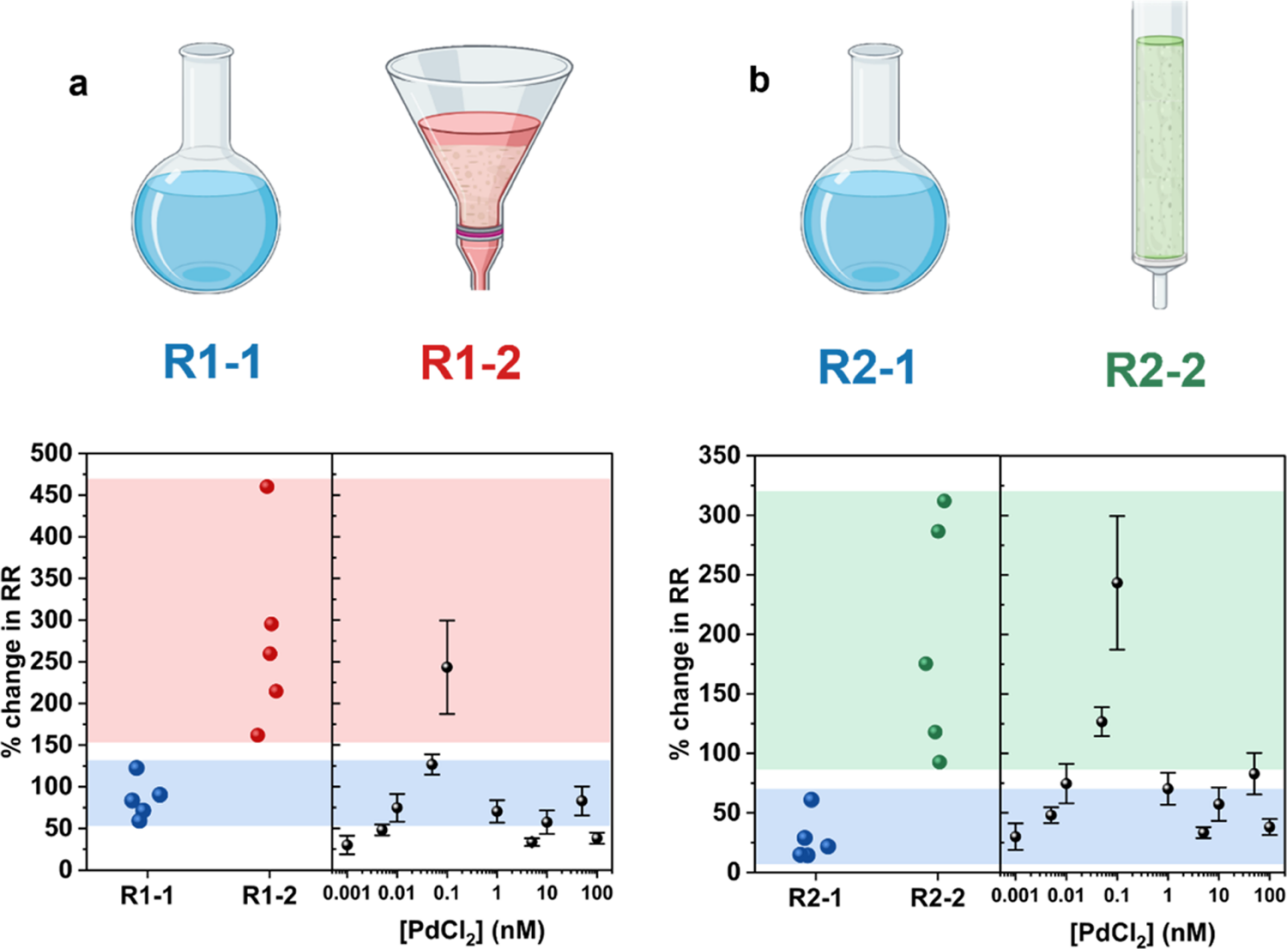
(a, b) Electrochemical response of 5 cyclam-functionalized nanopipettes to the organic product of (a) the Suzuki–Miyaura reaction (R1) before (R1-1) and after (R1-2) Celite filtration. Standard error calculations indicate an 85 ± 10% change for R1-1, and 278 ± 45% change for R1-2. (b) The same analysis for the DAAA reaction (R2) before (R2-1) and after (R2-2) column chromatography. Standard error calculations indicate a 28 ± 8% change for R2-1 and 196 ± 39% change for R2-2.

Figure 8a shows the results of the electrochemical analysis of the Suzuki–Miyaura reaction, where R1-1 denotes the crude product and R1-2 denotes the pure product after Celite filtration. Both R1-1 and R1-2 produce an ICR response, indicating the presence of Pd. The increase in response from R1-1 to R1-2 is indicative of a decrease in the Pd content, as evident in the PdCl_2_ calibration curve shown in Figure 3b. R1-1 produces an 85 ± 10% change, corresponding to a series of likely concentrations, 50, 10, or 1 nM, with a response closest to that of 50 nM PdCl_2_. On the contrary, the 278 ± 45% change of sample R1-2 is indicative of the presence of 0.1 nM Pd, at the maximum of the PdCl_2_ calibration curve. ICP-MS analysis of the samples indicated the presence of 31.65 nM Pd in R1-1 and 0.78 nM Pd in R1-2.

Figure 8b shows the electrochemical analysis of the DAAA reaction, where R2-1 denotes the crude product and R2-2 denotes the product after column chromatography. Similarly to the Suzuki–Miyaura reaction, both R2-1 and R2-2 produce an ICR response, indicating the presence of Pd, with an increase in response from R2-1 to R2-2 indicative of a decreasing concentration of Pd. R2-1 gives a 28 ± 8% change, and is determined to contain 100, 10, or 5 nM Pd, with an ICR response closest to that of 10 nM. R2-2 produces a response similar to R1-2 of 197 ± 39%, at the maximum of the PdCl_2_ calibration curve, indicating the presence of 0.1 nM Pd in the sample. ICP-MS analysis confirms the presence of 16.97 nM Pd in R2-1, and 0.14 nM Pd in R2-2, which agrees closely with our nanopore analysis.

These results demonstrate the viability of our sensor in “real world” applications and the importance of developing such devices, as metal ions are difficult to remove entirely from organic products. Our analysis can be carried out immediately without the requirement for extensive sample pretreatment like the concentrated acid digestion required for ICP-MS or OES, which is beneficial both practically and cost wise. Some further drawbacks of ICP-MS or OES include equipment and operating costs, as well as the high level of expertise required.^57^ Our sensor only requires simple electronic instrumentation, a low-cost easily fabricated nanopipette, and a polar aprotic solvent. Additionally, our sensor is operable in a relatively complex solution of an unpurified reaction product, giving results comparable to those obtained by ICP-MS of the digested sample. In the future, we will consider more complex media for analysis, such as products containing other interferent metal ions.

## Conclusions

To conclude, we have developed a simple, one-step dipping procedure for the functionalization of quartz nanopipettes with cyclam. The resulting nanopipettes can be used for the detection of trace amounts of metal species in aprotic solvent, based on changes in ICR. Finite element simulations are used to support our experimental findings, showing an oscillatory, nonlinear ICR response as a function of decreasing metal concentration. The devices have been used to determine the presence of Pd in organic products of Pd-catalyzed Suzuki–Miyaura and DAAA reactions before and after Celite filtration and column chromatography, with close agreement to ICP-MS analysis of the same samples. This work is the first example of ICR-based nanopore sensing in nonaqueous systems and can be used to determine trace metal residue postpurification in a synthetic pathway with no requirement for pretreatment. This paves the way for the development of low-cost, simple, in-line contaminant detection techniques based on nanoscale electrochemical phenomena.

## Supporting Information Available

Full experimental procedures, additional experimental data, and additional technical details regarding the developed COMSOL model (PDF)

## Abbreviations

ICR: ion current rectification
CV: cyclic voltammogram
MeCN: acetonitrile
ICP: inductively coupled plasma
MS: mass spectrometry
OES: optical emission spectroscopy
TEATFB: tetraethylammonium tetrafluoroborate

## References

[ref1] White H. S.; Bund A. Ion current rectification at nanopores in glass membranes. Langmuir 2008, 24 (5), 2212–2218. 10.1021/la702955k. 18225931

[ref2] Lan W. J.; Holden D. A.; White H. S. Pressure-Dependent Ion Current Rectification in Conical-Shaped Glass Nanopores. J. Am. Chem. Soc. 2011, 133 (34), 13300–13303. 10.1021/ja205773a. 21800889

[ref3] Woermann D. Electrochemical transport properties of a cone-shaped nanopore: high and low electrical conductivity states depending on the sign of an applied electrical potential difference. Phys. Chem. Chem. Phys. 2003, 5 (9), 1853–1858. 10.1039/b301021j.

[ref4] Siwy Z.; Fulinski A. A nanodevice for rectification and pumping ions. Am. J. Phys. 2004, 72 (5), 567–574. 10.1119/1.1648328.

[ref5] Siwy Z.; Heins E.; Harrell C. C.; Kohli P.; Martin C. R. Conical-nanotube ion-current rectifiers: The role of surface charge. J. Am. Chem. Soc. 2004, 126 (35), 10850–10851. 10.1021/ja047675c. 15339163

[ref6] Cervera J.; Schiedt B.; Ramirez P. A Poisson/Nernst-Planck model for ionic transport through synthetic conical nanopores. Europhys. Lett. 2005, 71 (1), 35–41. 10.1209/epl/i2005-10054-x.

[ref7] Cervera J.; Schiedt B.; Neumann R.; Mafe S.; Ramirez P. Ionic conduction, rectification, and selectivity in single conical nanopores. J. Chem. Phys. 2006, 124 (10), 104706 10.1063/1.2179797. 16542096

[ref8] Duleba D.; Johnson R. P. Sensing with ion current rectifying solid-state nanopores. Curr. Opin. Electrochem. 2022, 34, 100989 10.1016/j.coelec.2022.100989.

[ref9] Zhang S. J.; Chen W.; Song L. B.; Wang X. H.; Sun W. L.; Song P. Y.; Ashraf G.; Liu B.; Zhao Y. D. Recent advances in ionic current rectification based nanopore sensing: a mini-review. Sens. Actuators Rep. 2021, 3, 100042 10.1016/j.snr.2021.100042.

[ref10] Wang J.; Martin C. R. A new drug-sensing paradigm based on ion-current rectification in a conically shaped nanopore. Nanomedicine 2008, 3 (1), 13–20. 10.2217/17435889.3.1.13. 18393663

[ref11] Guo Z. J.; Wang J. H.; Wang E. K. Selective discrimination of small hydrophobic biomolecules based on ion-current rectification in conically shaped nanochannel. Talanta 2012, 89, 253–257. 10.1016/j.talanta.2011.12.022. 22284488

[ref12] Xie Z. P.; Yang M. F.; Luo L.; Lv Y. P.; Song K. J.; Liu S. M.; Chen D. Q.; Wang J. H. Nanochannel sensor for sensitive and selective adamantanamine detection based on host-guest competition. Talanta 2020, 219, 121213 10.1016/j.talanta.2020.121213. 32887115

[ref13] Vilozny B.; Wollenberg A. L.; Actis P.; Hwang D.; Singaram B.; Pourmand N. Carbohydrate-actuated nanofluidic diode: switchable current rectification in a nanopipette. Nanoscale 2013, 5 (19), 9214–9221. 10.1039/c3nr02105j. 23934399 PMC3827696

[ref14] Nguyen Q. H.; Ali M.; Neumann R.; Ensinger W. Saccharide/glycoprotein recognition inside synthetic ion channels modified with boronic acid. Sens. Actuators, B 2012, 162 (1), 216–222. 10.1016/j.snb.2011.12.070.

[ref15] Shi L.; Zhang Z. H.; Zhang L. M.; Tian Y. Electrochemical Detection of Tyrosinase in Cell Lysates at Functionalized Nanochannels via Amplifying of Ionic Current Response. Electroanalysis 2022, 34 (6), 1021–1026. 10.1002/elan.202100358.

[ref16] Zhang K. L.; He X. L.; Liu Y.; Yu P.; Fei J. J.; Mao L. Q. Highly Selective Cerebral ATP Assay Based on Micrometer Scale Ion Current Rectification at Polyimidazolium-Modified Micropipettes. Anal. Chem. 2017, 89 (12), 6794–6799. 10.1021/acs.analchem.7b01218. 28516771

[ref17] Ma W. H.; Xie W. Y.; Tian R.; Zeng X. Q.; Liang L. Y.; Hou C. J.; Huo D. Q.; Wang D. Q. An ultrasensitive aptasensor of SARS-CoV-2 N protein based on ion current rectification with nanopipettes. Sens. Actuators, B 2023, 377, 133075 10.1016/j.snb.2022.133075. PMC9700395 36467330

[ref18] Nasir S.; Ali M.; Ahmed I.; Niemeyer C. M.; Ensinger W. Phosphoprotein Detection with a Single Nanofluidic Diode Decorated with Zinc Chelates. ChemPlusChem 2020, 85 (3), 587–594. 10.1002/cplu.202000045. 32216097

[ref19] Cai S. L.; Cao S. H.; Zheng Y. B.; Zhao S.; Yang J. L.; Li Y. Q. Surface charge modulated aptasensor in a single glass conical nanopore. Biosens. Bioelectron. 2015, 71, 37–43. 10.1016/j.bios.2015.04.002. 25884732

[ref20] Duan L.; Yobas L. Label-Free Multiplexed Electrical Detection of Cancer Markers on a Microchip Featuring an Integrated Fluidic Diode Nanopore Array. ACS Nano 2018, 12 (8), 7892–7900. 10.1021/acsnano.8b02260. 30024729

[ref21] Zhang S. Q.; Sun T.; Wang J. H. Biomimetic phosphate assay based on nanopores obtained by immobilization of zirconium(IV) on a film of polyethyleneimine. Microchim. Acta 2015, 182 (7–8), 1387–1393. 10.1007/s00604-015-1459-y.

[ref22] Xie G. H.; Xiao K.; Zhang Z.; Kong X. Y.; Liu Q.; Li P.; Wen L. P.; Jiang L. A Bioinspired Switchable and Tunable Carbonate-Activated Nanofluidic Diode Based on a Single Nanochannel. Angew. Chem., Int. Ed. 2015, 54 (46), 13664–13668. 10.1002/anie.201505269. 26383001

[ref23] Fu Y. Q.; Tokuhisa H.; Baker L. A. Nanopore DNA sensors based on dendrimer-modified nanopipettes. Chem. Commun. 2009, (32), 4877–4879. 10.1039/b910511e. 19652811

[ref24] Ali M.; Neumann R.; Ensinger W. Sequence-Specific Recognition of DNA Oligomer Using Peptide Nucleic Acid (PNA)-Modified Synthetic Ion Channels: PNA/DNA Hybridization in Nanoconfined Environment. ACS Nano 2010, 4 (12), 7267–7274. 10.1021/nn102119q. 21082785

[ref25] Xiong Y. Z.; Ma T.; Zhang H.; Qiu L. Z.; Chang S.; Yang Y. W.; Liang F. Gold nanoparticle functionalized nanopipette sensors for electrochemical paraquat detection. Microchim. Acta 2022, 189 (7), 251 10.1007/s00604-022-05348-9. 35680710

[ref26] Zhao D. D.; Tang H. R.; Wang H.; Yang C.; Li Y. X. Analytes Triggered Conformational Switch of i-Motif DNA inside Gold-Decorated Solid-State Nanopores. ACS Sens. 2020, 5 (7), 2177–2183. 10.1021/acssensors.0c00798. 32588619

[ref27] Actis P.; Jejelowo O.; Pourmand N. Ultrasensitive mycotoxin detection by STING sensors. Biosens. Bioelectron. 2010, 26 (2), 333–337. 10.1016/j.bios.2010.08.016. 20829024 PMC2946441

[ref28] Zhang S. Q.; Li K. B.; Pan Y. J.; Han D. M. Ultrasensitive detection of ochratoxin A based on biomimetic nanochannel and catalytic hairpin assembly signal amplification. Talanta 2020, 220, 121420 10.1016/j.talanta.2020.121420. 32928431

[ref29] Zhan K.; Li Z. Y.; Chen J.; Hou Y. Q.; Zhang J.; Sun R. Q.; Bu Z. X.; Wang L. Y.; Wang M.; Chen X. Y.; Hou X. Tannic acid modified single nanopore with multivalent metal ions recognition and ultra-trace level detection. Nano Today 2020, 33, 100868 10.1016/j.nantod.2020.100868.

[ref30] Gao R.; Ying Y. L.; Yan B. Y.; Iqbal P.; Preece J. A.; Wu X. Y. Ultrasensitive determination of mercury(II) using glass nanopores functionalized with macrocyclic dioxotetraamines. Microchim. Acta 2016, 183 (1), 491–495. 10.1007/s00604-015-1634-1.

[ref31] Sa N. Y.; Fu Y. Q.; Baker L. A. Reversible Cobalt Ion Binding to Imidazole-Modified Nanopipettes. Anal. Chem. 2010, 82 (24), 9963–9966. 10.1021/ac102619j. 21090777 PMC3075968

[ref32] Zhai Q. F.; Wang J. H.; Jiang H.; Wei Q.; Wang E. K. Bare conical nanopore embedded in polymer membrane for Cr(III) sensing. Talanta 2015, 140, 219–225. 10.1016/j.talanta.2015.03.035. 26048845

[ref33] Chen L. Z.; He H. L.; Xu X. L.; Jin Y. D. Single glass nanopore-based regenerable sensing platforms with a non-immobilized polyglutamic acid probe for selective detection of cupric ions. Anal. Chim. Acta 2015, 889, 98–105. 10.1016/j.aca.2015.06.051. 26343431

[ref34] Actis P.; Vilozny B.; Seger R. A.; Li X.; Jejelowo O.; Rinaudo M.; Pourmand N. Voltage-Controlled Metal Binding on Polyelectrolyte-Functionalized Nanopores. Langmuir 2011, 27 (10), 6528–6533. 10.1021/la2005612. 21510657 PMC3099346

[ref35] Heaton I.; Platt M. Multiuse Nanopore Platform with Disposable Paper Analytical Device for the Detection of Heavy Metal Ions. Ind. Eng. Chem. Res. 2020, 59 (49), 21403–21412. 10.1021/acs.iecr.0c04806.

[ref36] Mo R. J.; Yuan Q.; Yan X. M.; Su T. T.; Feng Y. T.; Lv L. L.; Zhou C. X.; Hong P. Z.; Sun S. L.; Li C. Y. A Mercury Ion Electrochemical Sensor Based on Porous Anodized Alumina Membrane Nanochannels Modified with DNA. J. Electrochem. Soc. 2018, 165 (11), H750–H755. 10.1149/2.1021811jes.

[ref37] Busacca C. A.; Fandrick D. R.; Song J. J.; Senanayake C. H. The Growing Impact of Catalysis in the Pharmaceutical Industry. Adv. Synth. Catal. 2011, 353 (11–12), 1825–1864. 10.1002/adsc.201100488.

[ref38] Constable D. J. C.; Jimenez-Gonzalez C.; Henderson R. K. Perspective on solvent use in the pharmaceutical industry. Org. Process Res. Dev. 2007, 11 (1), 133–137. 10.1021/op060170h.

[ref39] Plett T.; Shi W. Q.; Zeng Y. H.; Mann W.; Vlassiouk I.; Baker L. A.; Siwy Z. S. Rectification of nanopores in aprotic solvents - transport properties of nanopores with surface dipoles. Nanoscale 2015, 7 (45), 19080–19091. 10.1039/C5NR06340J. 26523891

[ref40] Yin X. H.; Zhang S. D.; Dong Y. T.; Liu S. J.; Gu J.; Chen Y.; Zhang X.; Zhang X. H.; Shao Y. H. Ionic Current Rectification in Organic Solutions with Quartz Nanopipettes. Anal. Chem. 2015, 87 (17), 9070–9077. 10.1021/acs.analchem.5b02337. 26218167

[ref41] Polster J. W.; Souna A. J.; Motevaselian M. H.; Lucas R. A.; Tran J. D.; Siwy Z. S.; Aluru N. R.; Fourkas J. T. The electrical-double layer revisited. Nat. Sci. 2022, 2 (2), 20210099 10.1002/ntls.20210099.

[ref42] Souna A. J.; Motevaselian M. H.; Polster J. W.; Tran J. D.; Siwy Z. S.; Aluru N. R.; Fourkas J. T. Beyond the electrical double layer model: ion-dependent effects in nanoscale solvent organization. Phys. Chem. Chem. Phys. 2024, 26, 6726 10.1039/D3CP05712G. 38323484

[ref43] Silva S.; Singh S.; Cao E.; Fourkas J. T.; Siwy Z. S. Gating ion and fluid transport with chiral solvent. Faraday Discuss. 2023, 246 (0), 508–519. 10.1039/D3FD00063J. 37427451

[ref44] Farrell E. B.; Duleba D.; Johnson R. P. Aprotic Solvent Accumulation Amplifies Ion Current Rectification in Conical Nanopores. J. Phys. Chem. B 2022, 126 (30), 5689–5694. 10.1021/acs.jpcb.2c03172. 35867912 PMC9358645

[ref45] Zalesskiy S. S.; Ananikov V. P. Pd_2_(dba)_3_ as a Precursor of Soluble Metal Complexes and Nanoparticles: Determination of Palladium Active Species for Catalysis and Synthesis. Organometallics 2012, 31 (6), 2302–2309. 10.1021/om201217r.

[ref46] Del Linz S.; Willman E.; Caldwell M.; Klenerman D.; Fernandez A.; Moss G. Contact-Free Scanning and Imaging with the Scanning Ion Conductance Microscope. Anal. Chem. 2014, 86 (5), 2353–2360. 10.1021/ac402748j. 24521282 PMC3944807

[ref47] Perry D.; Momotenko D.; Lazenby R. A.; Kang M.; Unwin P. R. Characterization of Nanopipettes. Anal. Chem. 2016, 88 (10), 5523–5530. 10.1021/acs.analchem.6b01095. 27108872

[ref48] James J.; Guiry P. J. Highly Enantioselective Construction of Sterically Hindered α-Allyl-α-Aryl Lactones via Palladium-Catalyzed Decarboxylative Asymmetric Allylic Alkylation. ACS Catal. 2017, 7 (2), 1397–1402. 10.1021/acscatal.6b03355.

[ref49] Feng G.; Huang J. S.; Sumpter B. G.; Meunier V.; Qiao R. Structure and dynamics of electrical double layers in organic electrolytes. Phys. Chem. Chem. Phys. 2010, 12 (20), 5468–5479. 10.1039/c000451k. 20467670

[ref50] Dubois G.; Tripier R.; Branes S. P.; Denat F.; Guilard R. Cyclam complexes containing silica gels for dioxygen adsorption. J. Mater. Chem. 2002, 12 (8), 2255–2261. 10.1039/b203372k.

[ref51] Dubois G.; Corriu R. J. P.; Reye C.; Brandes S.; Denat F.; Guilard R. First organic-inorganic hybrid materials with controlled porosity incorporating cyclam units. Chem. Commun. 1999, (22), 2283–2284. 10.1039/a907217i.

[ref52] Martin R.; Buchwald S. L. Palladium-Catalyzed Suzuki-Miyaura Cross-Coupling Reactions Employing Dialkylbiaryl Phosphine Ligands. Acc. Chem. Res. 2008, 41 (11), 1461–1473. 10.1021/ar800036s. 18620434 PMC2645945

[ref53] Pàmies O.; Margalef J.; Cañellas S.; James J.; Judge E.; Guiry P. J.; Moberg C.; Bäckvall J. E.; Pfaltz A.; Pericàs M. A.; Diéguez M. Recent Advances in Enantioselective Pd-Catalyzed Allylic Substitution: From Design to Applications. Chem. Rev. 2021, 121 (8), 4373–4505. 10.1021/acs.chemrev.0c00736. 33739109 PMC8576828

[ref54] James J.; Jackson M.; Guiry P. J. Palladium-Catalyzed Decarboxylative Asymmetric Allylic Alkylation: Development, Mechanistic Understanding and Recent Advances. Adv. Synth. Catal. 2019, 361 (13), 3016–3049. 10.1002/adsc.201801575.

[ref55] Akula R.; Doran R.; Guiry P. J. Highly Enantioselective Formation of α-Allyl-α-Arylcyclopentanones via Pd-Catalysed Decarboxylative Asymmetric Allylic Alkylation. Chem. – Eur. J. 2016, 22 (29), 9938–9942. 10.1002/chem.201602250. 27191198

[ref56] Jackson M.; O’Broin C. Q.; Müller-Bunz H.; Guiry P. J. Enantioselective synthesis of sterically hindered α-allyl-α-aryl oxindoles palladium-catalysed decarboxylative asymmetric allylic alkylation. Org. Biomol. Chem. 2017, 15 (38), 8166–8178. 10.1039/C7OB02161E. 28920985

[ref57] Wilschefski S. C.; Baxter M. R. Inductively Coupled Plasma Mass Spectrometry: Introduction to Analytical Aspects. Clin. Biochem. Rev. 2019, 40 (3), 115–133. 10.33176/AACB-19-00024. 31530963 PMC6719745

