# Supplementary material for: The Detection of Trace Metal Contaminants in Organic Products Using Ion Current Rectifying Quartz Nanopipettes
Source: Anal Chem. 2024 Apr 3;96(15):6055–64. doi: 10.1021/acs.analchem.4c00634 (PMC11024892; doi:10.1021/acs.analchem.4c00634)
Supplement: Supplementary file 1 — ac4c00634_si_001.pdf [file ac4c00634_si_001.pdf]

Supporting Information:
The Detection of Trace Metal Contaminants in Organic Products Using Ion Current Rectifying Quartz Nanopipettes

Emer B. Farrell, Fionn McNeill, Alexander Weiss, Dominik Duleba, Patrick J. Guiry, Robert P. Johnson*

School of Chemistry, University College Dublin, Belfield, Dublin 4, Ireland

*robert.johnson@ucd.ie

Contents

1. Finite Element Simulations

2. Nanopipette Characterization

3. Characterization of 3-(1,4,8,11-tetraazacyclotetradecane)propyltrimethoxysilane (silyl cyclam)

4. Reusability Studies of Cyclam-Functionalized Nanopipette Sensors for Pd Detection

1. Finite Element Simulations:

Capillary Filling Model Background and Equations:

The interface-tension σ_{ij} and the contact angle θ are two parameters governing the shape of a meniscus via the law of capillary action;

$$\sigma_{13} - \sigma_{23} = \sigma_{12}\cos\theta$$

(S1)

where the indices $i, j \in \{1, 2, 3\}$ denote the gas, liquid, and solid phase, respectively.

The height of the meniscus h is then given by Jurin's law.

$$\mathrm{h} = \frac{2\sigma\cos\theta}{\rho \mathrm{gr}}$$

(S2)

which is derived by equating the capillary force $F_C = 2\pi r\sigma$ and the gravitational force of the liquid $G = \rho\pi r^2 hg$ and substituting the surface tension σ with the contact angle.

The Cahn-Hilliard equations were solved to track the diffuse interface with the phase field help variable, Ψ.

$$\frac{\partial\phi}{\partial t} + u \cdot \nabla\phi = \nabla \cdot \frac{\gamma\lambda}{\varepsilon^2} \nabla\Psi$$
$$\Psi = -\nabla \cdot \varepsilon^2 \nabla\phi + (\Phi^2 - 1)\phi$$

(S3)

where u is the fluid velocity in m s^{-1}, γ is the mobility in m^3 s kg^{-1}, λ is the mixing energy density in N and ε is the interface thickness parameter in m.
For the surface tension coefficient σ follows:

$$\sigma = \frac{2\sqrt{2}}{3}\frac{\lambda}{\varepsilon}$$

(S4)

and the volume fractions of the two phases are given by:

$$\mathrm{V}(\phi) = \frac{(1 + \phi)}{2} V_1 + \frac{(1 - \phi)}{2} V_2$$

The physical properties of the fluids such as the density in kg m^{-3} and the viscosity μ in Pa s are then interpolated between the two phases using:

$$\rho = \rho_l + (\rho_g - \rho_l)V_2$$
$$\mu = \mu_l + (\mu_g - \mu_l)V_2$$

(S5)

To account for mass and momentum transport the Navier-Stokes equations are included in the model-physics with the surface tension force acting at the interface F_{st}:

$$\rho \frac{\partial u}{\partial t} + \rho(u \cdot \nabla)u = \nabla \cdot [-pI + \mu(\nabla u + (\nabla u)^T)] + F_{st} + \rho g$$
$$\text{and } \nabla \cdot u = 0 \qquad (S6)$$

where ρ is the density in kg m^{-3} , μ is the dynamic viscosity in Ns m^{-2} , u is the velocity in m s^{-1}, p is the pressure in Pa and g is the gravity vector in m s^{-2}.

Using the chemical potential G in J m^3:

$$G = \lambda \left[-\nabla^2\phi + \frac{(\phi^2 - 1)}{\varepsilon^2} \right] = \frac{\lambda}{\varepsilon^2} \Psi \qquad (S7)$$

the surface tension can be calculated only with Ψ and the gradient of the phase field variable,

$$F_{st} = G\nabla\phi \qquad (S8)$$

avoiding the usage of the surface normal and the surface curvature. The model-physics are adapted from a model example published by COMSOL® in their model library (application ID 1878).

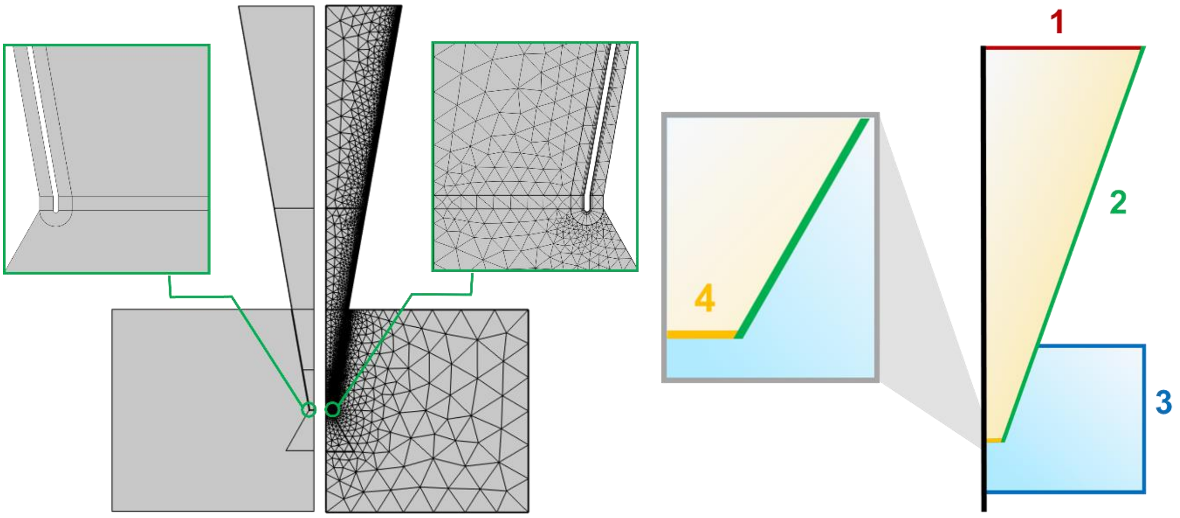

Figure S1. The 2D axisymmetric geometry and corresponding mesh of a 50 nm quartz nanopipette used in finite element simulations, with boundaries corresponding to the boundary conditions in Table S1 highlighted.

Table S1. The boundary conditions employed in finite element simulations of capillary filling and ion current rectification in 50 nm quartz nanopipettes, in MeCN, corresponding to the 2D axisymmetric geometry shown in Figure S1.

**Ion Current Rectification Model**			
**Boundary**	**Nernst Planck (tds)**	**Navier Stokes (spf)**	**Poisson (es)**
**1. Interior Bulk**	Concentration<br>cbulk	Inlet<br>Pressure, 1 atm	Applied Potential
**2. Nanopipette Wall**	No Flux	No Slip	Surface Charge
**3. Exterior Bulk**	Concentration<br>cbulk	Outlet<br>Pressure, 1 atm	Ground
**Capillary Filling Model**			
**Boundary**	**Phase Field (pf)**	**Navier Stokes (spf)**	-
**1. Interior Bulk**	Outlet	Inlet<br>Pressure, 1 atm	-
**2. Nanopipette Wall**	Specified θ via equation S1	No Slip	-
**3. Exterior Bulk**	Inlet, Fluid 2 (ϕ = 1)	Outlet<br>Pressure, 1 atm	-
**4. Phase Transition**	Initial Interface	-	-

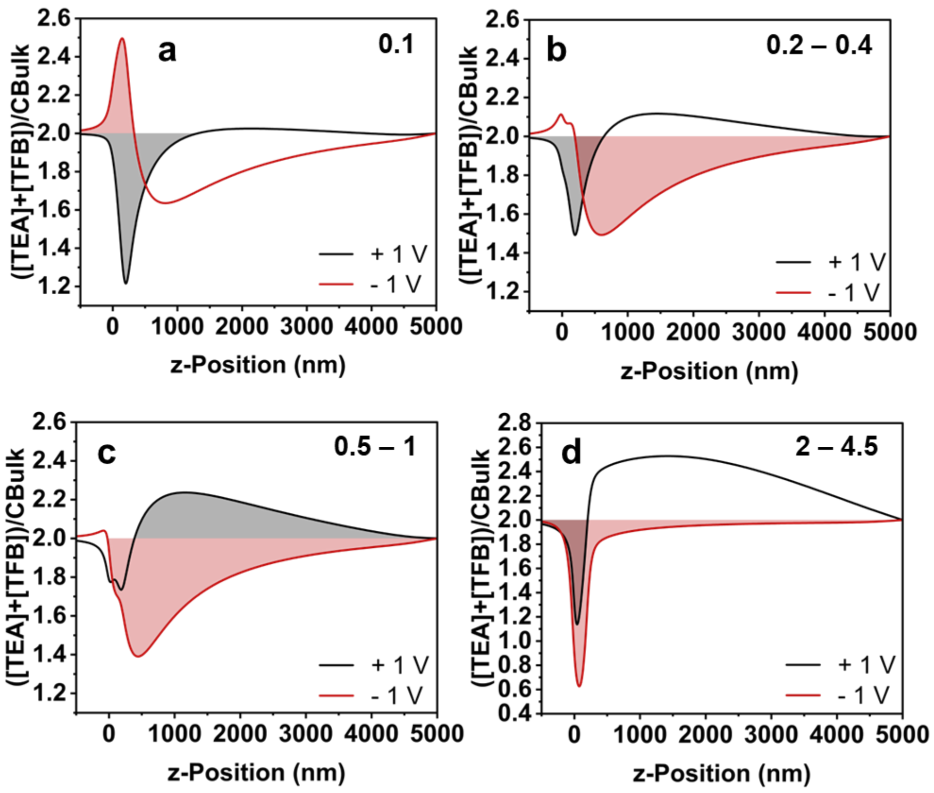

Figure S2: Normalized ion enrichment and depletion bands obtained at + 1 and -1 V along the *z*-axis, with surface charge densities of a) 0.1 mC m^{-2}, b) 0.2 – 0.4 mC m^{-2}, c) 0.5 – 1 mC m^{-2}, and d) 2 – 4.5 mC m^{-2}, in the cyclam functionalized region of the nanopipette tip (0.1 – 4.5 mC m^{-2}). The shaded regions are included in the model as accumulation or depletion diodes, and are summarized in Table S2.

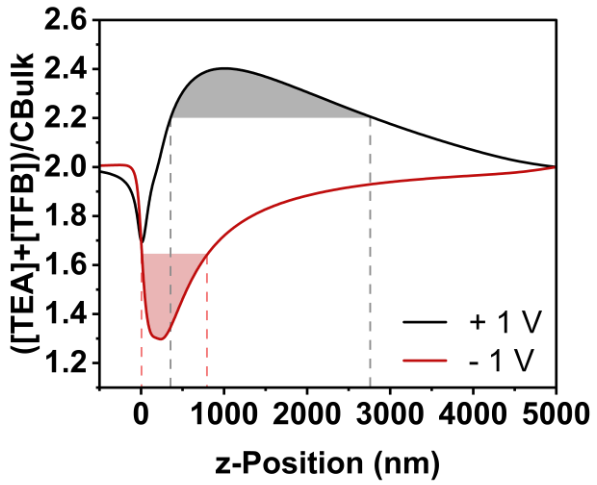

Figure S3. The procedure employed for selecting diode boundaries from the half peak height of ion enrichment and depletion bands. The boundaries are summarised in Table S2.

Table S2. Summarized state (accumulation or depletion) and boundaries (obtained as per procedure in Figure S3) of double junction diodes as a function of increasing surface charge density in the cyclam functionalized region of a 50 nm quartz nanopipette.

Surface Charge Density in Cyclam-Functionalized Region ($mC\ m^{-2}$)	Diode Boundaries (+ 1 V)	State of Diode (+ 1 V)	Diode Boundaries (– 1 V)	State of Diode (– 1 V)
0.1	84 - 405	depletion	0 - 250	accumulation
0.2	78 - 379	depletion	400 - 1575	depletion
0.3	61 - 355	depletion	290 - 1720	depletion
0.4	0 - 323	depletion	230 - 1530	depletion
0.5	535 - 2845	accumulation	130 - 1325	depletion
0.6	400 - 2800	accumulation	45 - 1120	depletion
0.7	400 - 2800	accumulation	22 - 970	depletion
0.8	355 - 2800	accumulation	10 - 813	depletion
0.9	326 - 2794	accumulation	3 - 660	depletion
1	295 - 2855	accumulation	0 - 514	depletion
2	0 – 117	depletion	0 - 230	depletion
3	0 - 140	depletion	0 -200	depletion
3.5	0 - 150	depletion	0-200	depletion
4	0 - 150	depletion	0-200	depletion
4.5	0 - 150	depletion	0-200	depletion

2. Nanopipette Characterization

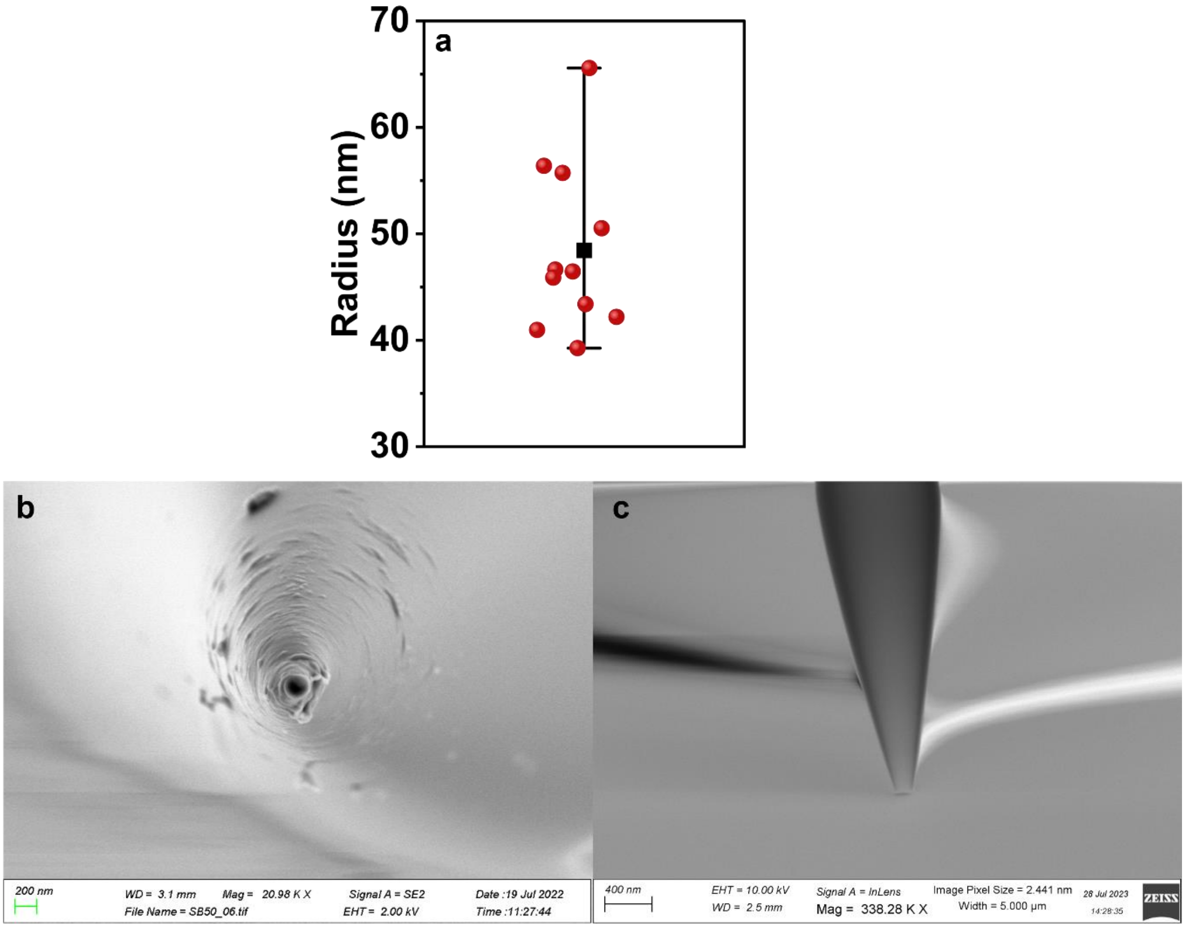

Figure S4. Nanopipette radius determination using **a)** conductivity measurements indicating a mean radius of 48.5 nm. **b,c)** Representative SEM and STEM images indicating an internal radius of ~ 60 nm.

3. Characterization of 3-(1,4,8,11-tetraazacyclotetradecane)propyltrimethoxysilane (silyl cyclam)

Mass Spectrometry:

Mass spectra of silyl cyclam (0.1 mg mL^{-1} in MeCN) were recorded on an Agilent 6546 Q-TOF instrument with an isocratic MeOH mobile phase, indicating the presence of a $C_{32}H_{74}N_8O_6Si_2$ species. This corresponds to a dimerized form of the target compound, as shown in Figure S5.

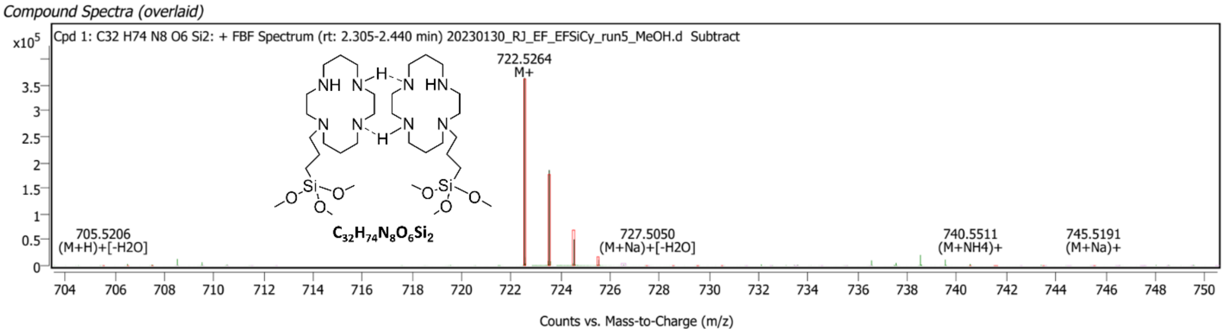

Figure S5. MS of 3-(1,4,8,11-tetraazacyclotetradecane)propyltrimethoxysilane (silyl cyclam), indicating the presence of a $C_{32}H_{74}N_8O_6Si_2$ species, which is hypothesized to belong to the as shown hydrogen bonded dimerized species.

4. Reusability Studies of Cyclam-Functionalized Nanopipette Sensors for Pd Detection

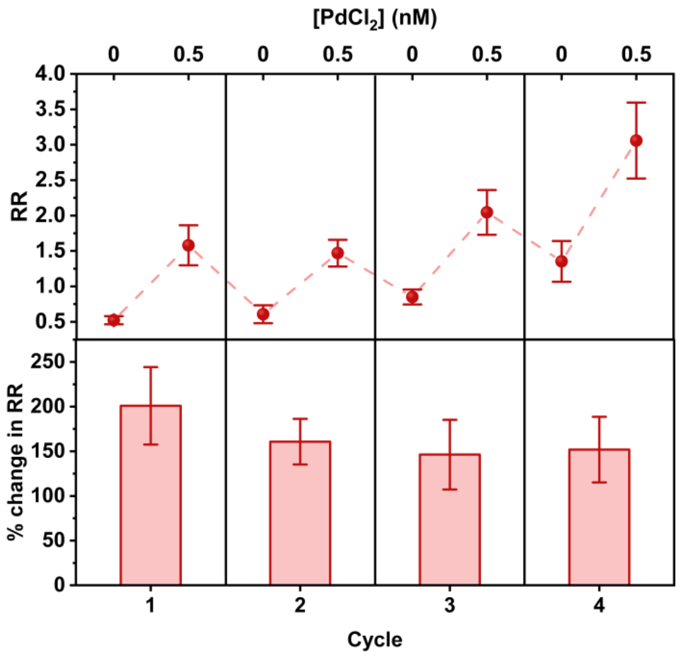

Figure S6. Reusability studies of 6 cyclam functionalized 50 nm quartz nanopipettes in the presence of 0 and 0.5 nM PdCl2 in the bulk external electrolyte solution. Nanopipettes are reused by heating on a hot plate surface at 60 °C for 50 min. At cycle 4, the RR of the cyclam functionalized nanopipettes inverts to ∼ 1, indicating a change in the surface state, but average percentage change remains within the statistical margin of error.
